# A concept for trial institutions focussing on randomised controlled trials in surgery

**DOI:** 10.1186/1745-6215-9-3

**Published:** 2008-01-24

**Authors:** Nuh N Rahbari, Markus K Diener, Lars Fischer, Moritz N Wente, Peter Kienle, Markus W Büchler, Christoph M Seiler

**Affiliations:** 1 Department of General, Visceral and Trauma Surgery, University of Heidelberg, Im Neuenheimer Feld 110, 69120 Heidelberg, Germany

## Abstract

**Background:**

Although considered the reference standard for generating valid scientific evidence of a treatment's benefits and harms, the number of Randomised Controlled Trials (RCT) comparing surgical techniques remains low. Much effort has been made in order to overcome methodological issues and improve quality of RCTs in surgery. To the present there has been, however, only little emphasis on development and maintenance of institutions for implementation of adequately designed and conducted surgical RCTs.

**Mehods/Design:**

Description of the developments in surgical RCT infrastructure in Germany between 2001 and 2006. Cross sectional evaluation of completed and ongoing surgical RCTs within the German Surgical Society and the Clinical Study Centre, Department of Surgery, University of Heidelberg.

**Results:**

Foundation of a national Clinical Trial Centre (CTC) for the organisation of multi-centre RCTs in the surgical setting (Study Center of the German Surgical Society, SDGC). Establishment of a network of CTCs with affiliated Clinical Sites (CSs) to enhance patient recruitment and shorten the duration of RCTs. Since its foundation four surgical RCTs with a total sample size of 1650 patients (1006 of these randomised) have been supervised by the SDGC with 35 CSs involved in patient recruitment. Five further CTCs were set up in 2006. Together with their affiliated CSs a network has been organised providing improved conditions for the conduction of surgical RCTs.

**Conclusion:**

Improvement of infrastructure substantially facilitates integration of RCTs into routine surgical practice. A network of collaborating CTCs and CSs can provide an adequate infrastructure for the conduction of multi-centre RCTs.

## Background

Randomised Controlled Trials (RCT) are considered the reference standard for generating valid scientific evidence on benefits and harms of treatments in surgery [1]. However, the percentage of RCTs dealing with surgical interventions is still low. A Medline analysis revealed that only about 15% of all published RCTs were conducted in the surgical setting [2]. It is estimated that only 24% of surgical therapies are based on the results of RCTs [3].

There are considerable national differences in the extent to which surgical RCTs (sRCTs) are pursued. In the United States the American College of Surgeons Oncology Group (ACOSOG) was founded in 1996 and is funded by the National Cancer Institute in order to increase the quantity and quality of sRCTs [4]. In Europe, the number of RCTs (adjusted for population size) conducted in the United Kingdom and the Netherlands is substantially higher than in most other European countries, with Germany lagging far behind [5]. Furthermore, the number of sRCTs in the leading German surgical journal "Der Chirurg" have decreased since 2000, while the corresponding number of sRCTs in international surgical journals has remained constant [6]. Although the German Science Council already recognised a lack of patient-oriented clinical research in Germany in the early 1990s, major national problems, i.e. lack of funding and a weak infrastructure for the implementation of surgical trials, were still present in 2003 [7]. However, within the last few years much effort has been invested into forming a working national infrastructure in surgical departments and the German Surgical Society (Deutsche Gesellschaft für Chirurgie) in order to establish an environment in which high-quality sRCTs can be performed on a larger scale.

The objective of this article is to describe recent developments in surgical trial infrastructure in Germany providing a sketch of how trial institutions for sRCTs might be organised. It's outline follows a previously proposed structure for reports dealing with quality improvement in health care [8].

### The problem

RCTs in surgery comprise several challenges regarding methodology (difficulties with standardisation and blinding), ethics (correct timing of sRCTs, clinical equipoise, and placebo surgery), surgeons (scepticism to randomise patients, lack of methodological expertise and time), and patients (preference for less invasive procedures, reluctance to be randomised to different surgical procedures) [1,9]. Therefore increasing the quantity and quality of sRCTs is a complex task requiring both a working infrastructure and sufficient funding.

### Key measures for improvement

A dual concept has been developed consisting of a Clinical Trial Centre (CTC), responsible for planning, conduction (management), and analysis of multi-centre sRCTs and of Clinical Sites (CS), which primarily focus on recruitment and treatment of study patients in accordance with Good Clinical Practice (GCP) guidelines [10]. The effectiveness of this approach can be evaluated quantitatively at the CTC level according to the number of performed multi-centre sRCTs, the number of recruited CSs, and the foundation of additional CTCs. In addition to these quantitative measures, established processes are evaluated qualitatively (e.g. the extent of collaborations with different scientific institutions and the adherence to the principles of GCP). Similarily, CSs are evaluated by the number and spectrum of conducted trials, the number of included patients, and the extent of cooperations.

### Gathering information and strategies for change

#### Clinical Trial Centre (CTC)

The development of a national CTC for multi-centre sRCTs was based on several meetings of the German Surgical Society's Steering Committee and a visit to ACOSOG. This visit was accompanied by representatives of the German Federal Ministry of Education and Research which constitutes the central national body for institutional grants and regularly announces new programs for the medical sciences. As a result thereof the German Surgical Society commissioned the Department of Surgery at the University of Heidelberg to apply for a grant in 2003 in order to establish the Study Centre of the German Surgical Society [Studienzentrum der Deutschen Gesellschaft für Chirurgie (SDGC)]. This decision was based on the pre-existing facilities for surgical trials and local expertise at the University of Heidelberg, including a well established Institute for Biometrics and a centre for clinical trials (see below). The aim of the SDGC is to provide services to surgeons intending to initiate or participate in multi-centre sRCTs. The tasks of the SDGC include: selection of trial ideas and study development, fund raising, registration of trials, compliance with legal requirements, ethical review management, conduction and analysis of trials, selection, monitoring, and auditing of CSs, quality assurance, education, and publication. An organisational structure was set up for the SDGC consisting of boards and units that involve representatives of the German Surgical Society and the Medical Faculty of the University of Heidelberg [9]. In order to properly address all relevant aspects of sRCTs, an interdisciplinary team (n = 12) consisting of surgeons trained in clinical trials, biometricians, study nurses, clinical research associates, data managers, monitors, and administrative people was formed. Furthermore, collaborations with relevant institutions were initiated, such as with the German Cochrane Centre for systematic reviews and meta-analysis (planning of trials) and the Coordinating Centres for Clinical Trials Network. Trial ideas can be submitted via the SDGC homepage and subsequently run through a four-step selection process which for approved studies ultimately results in a finalised study protocol. The SDGC prioritises study ideas following the *FINER *criteria: feasible, interesting, novel, ethical, relevant. A detailed description of the process with further details on study selection and protocol design was published by the SDGC [11].

The SDGC's CSs were formed based on the results of a nationwide survey of surgical departments during which 237 of 1274 departments expressed their willingness to participate in clinical trials.

The German Federal Ministry of Education and Research approved the grant to the SDGC after external peer review and funding started in 2005 for a period of six years. Additional funding was provided by the German Surgical Society and industrial partners (Ethicon, Tyco Healthcare, and Aesculap).

#### Clinical Sites (CS)

CSs provide access to patient populations. During multi-centre surgical trials, the duties of CSs comprise screening of patients for eligibility to be included in ongoing trials based on predefined inclusion criteria, informing and obtaining informed consent of patients, and treatment of patients according to a study protocol and to GCP. The Centre for Clinical Studies [Klinisches Studienzentrum Chirurgie (KSC)] at the Department of Surgery at the University of Heidelberg provides an example of how a CS can be set up [12]. The KSC was already founded in October 2001 with the intention of performing surgical trials in a standardised fashion and to increase the number of patients participating in surgical trials. After the recruitment of professional personnel (two surgeons, three study nurses, and a study secretary), the first patient was recruited into a sRCT in May 2002. Subsequently, a network between the Central Patient Management, responsible for all admissions to the Department of Surgery [13], the local ethics committee, and the hospital administration was established. This network ensures high efficiency in patient recruitment and treatment according to GCP as all patients admitted to the Department of Surgery are screened for eligibility for ongoing trials. Furthermore, time intervals for ethics approvals and finalising contracts could be substantially shortened. Recruitment of study patients was further facilitated by arranging for an on-call service which allows recruitment of patients at all times and is thus particularly helpful for the implementation of emergency trials. In order to ease funding constraints for surgical trials pharmacological studies were also pursued at the KSC. Moreover, pharmaceutical trials provide valuable experience and serve as examples for the design of high-quality surgical and investigator driven trials. As a CS of the SDGC the KSC participates in all multi-centre sRCTs, in addition to the single-centre sRCTs it performs.

### Effects of change

#### Clinical Trial Centers

Four multi-centre RCTs comparing surgical interventions (total sample size: 1650) have been initiated (open to patient recruitment) by the SDGC since 2004 (Table 1). The first trial (INSECT – interrupted versus continuous slowly absorbable sutures – evaluation of abdominal closure techniques) finished patient recruitment (n = 624) in October 2006. All study protocols were registered and have either been published or submitted for publication [14,15]. The number of CSs recruited and initiated for the SDGC's multi-centre sRCTs has now increased to 35. Two positions were created at the SDGC in order to teach surgical residents the methods of clinical research and epidemiology. In addition, a professional education program for surgeons in trials was successfully established, and the results of the participant evaluation published [16].

**Table 1:** Features of single-centre sRCTs (KSC) and multi-centre sRCTs (SDGC) (09/2007)

	Trial/ISRCTN	Interventions	Primary Endpoint	Sample size/Randomised patients/Clinical Sites
KSC	BEGER/BERN 50638764 [17]	Beger vs Berne procedure for surgical treatment of chronic pancreatitis	Duration of surgery Quality of life Time at ICU Duration of hospital stay	65/65
	POUCH 78983587	Transverse Coloplasty versus J-Pouch for low anterior rectal resection	Difficulties with evacuation after 2 years	150/150
	POVATI 60734227 [18]	Midline vs transverse laparotomy	Pain and use of analgesics	200/200
	LAPCON-POUCH 61411448 [19]	Laparoscopic vs. open total proctocolectomy with ileo-anal pouch anastomosis	Blood loss Units packed cells	130/30
	PORTAS 52368201 [20]	Venae Sectio vs. modified Seldinger Technique for Totally Implantable Access Ports	Primary success rate of surgical technique	164/164

SDGC	INSECT 24023541 [14]	Running vs interrupted fascia closure after midline laparotomy	Incisional hernia	600/624/25
	CLIVIT 96901396 [15]	Clips vs ligation in thyroid surgery	Duration of surgery	400/265/5
	DISPACT 18452029	Stapling vs scalpel transsection and hand-suture for distal pancreatectomy	Pancreatic fistula and mortality	550/101/25
	TOPAR-PILOT 86202793 [21]	Autotransplantation vs none after total parathyroidectomy	Recurrence of secondary hyperparathyroidism	100/16/7

ISRCTN = International Standard Randomised Controlled Trial Number

*BEGER/BERN *Duodenum preserving pancreatectomy in chronic pancreatitis: A randomised controlled trial comparing two surgical techniques; *POUCH *Colon J-Pouch versus Transverse Coloplasty Pouch after low anterior resection for rectal cancer: a randomised controlled trial; *POVATI *Postsurgical pain outcome of vertical and transverse abdominal incision – A randomised controlled equivalence trial; *PORTAS *Comparison of Venae Sectio vs. modified Seldinger Technique for Totally Implantable Access Ports; *LAPCON-POUCH *Totally laparoscopic vs conventional ileo-anal pouch procedure – design of a single-centre, expertise based randomised controlled trial to compare the laparoscopic and conventional surgical approach in patients undergoing primary elective restorative proctocolectomy; *INSECT *Interrupted vs continuous slowly absorbable sutures – evaluation of abdominal closure techniques; *CLIVIT *Clips vs ligature in thyroid surgery – a randomised controlled trial; *DISPACT *Distal pancreatectomy – A randomised controlled trial to compare to different surgical techniques; *TOPAR-Pilot *Secondary hyperparathyroidism: does total parathyroidectomy alone lead to lower rate of recurrence than total parathyroidectomy with autotransplantation? – A randomised controlled pilot trial.

**Figure 1:**
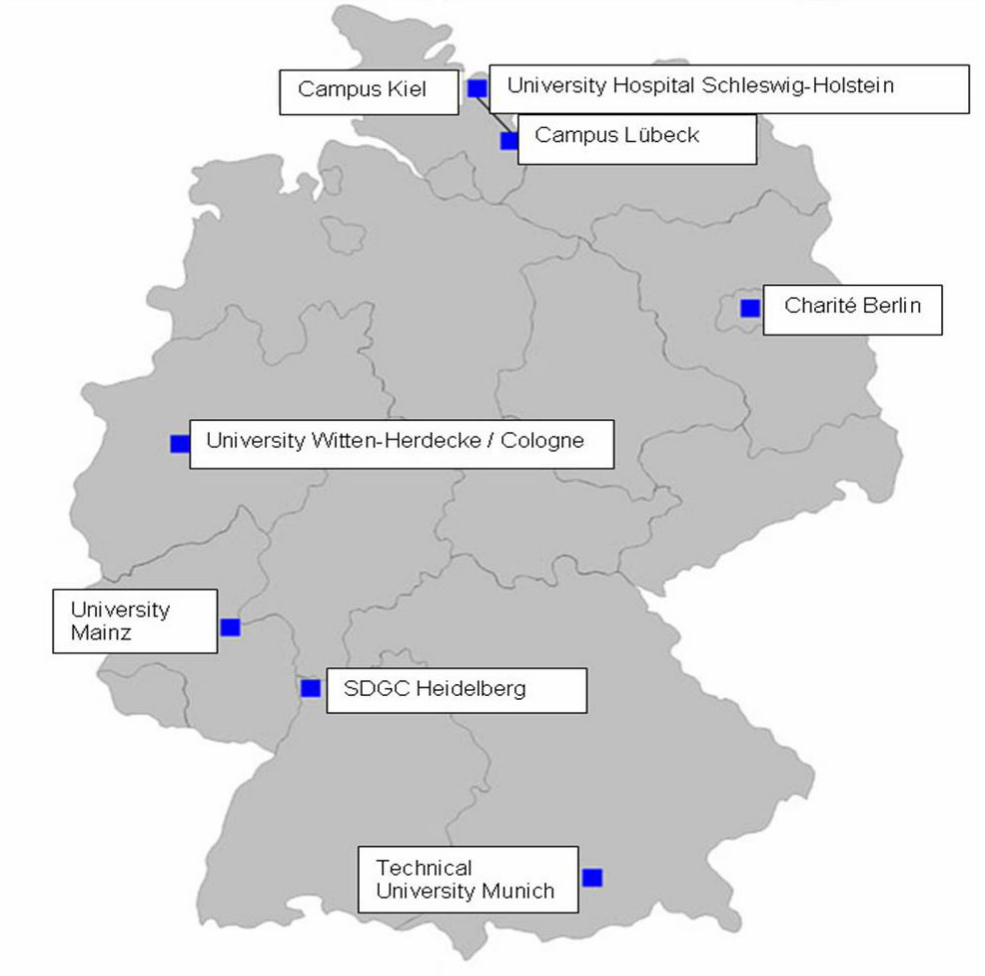
Clinical Trial Centres involved in the German Surgical Trial Network (CHIR-NET).

The number of multi-centre sRCTs and the time needed to accomplish the trials are highly dependent on the number of participating CTCs and the resources available. Based on the work of the SDGC which showed that it was feasible to integrate implementation of multi-centre trials into the clinical routine of surgical departments, if properly organised, a further grant program was announced by the German Federal Ministry for Education and Research in 2005 in order to support the installation of additional surgical CTCs. Surgical Departments of 22 Medical Faculties applied and five were selected after external peer review (Figure 1). In 2006 these five surgical CTCs and the SDGC formed the German Surgical Trial Network [Chirurgisches Netzwerk für operative Studien (CHIR-NET)]. Based on the requirement to cooperate with local academic institutions experienced in biometrics or clinical epidemiology CTCs of the CHIR-NET are supposed to design and coordinate multi-centre sRCTs which in turn will be supported by all members of the network. In addition each CTC cooperates with several CSs providing excellent access to patients. Training and education of study personnel and surgical residents is an additional joint task of the CHIR-NET's CTCs. As a result of this collaboration six large trial protocols have been submitted to the joint grant program for multi-centre RCTs of the German Research Foundation and the German Federal Ministry of Education and Research in November 2006.

#### Clinical Sites

Since its foundation in 2001, 25 clinical studies were successfully completed at the KSC (five surgical, two observational and 18 pharmaceutical studies). Currently, 24 clinical studies are supervised by the KSC (ten surgical, two observational and 12 pharmaceutical studies). To date, more than 1700 patients have been randomised and treated according to study protocols (Table 2). Five single-centre sRCTs have been set up, registered, and four out of five study protocols were published (Table 1) [17-20]. Results for the primary endpoint of three trials (Beger/Bern, POVATI, PORTAS) will be available in 2008.

**Table 2:** Current number of studies and study patients at the Clinical Study Centre Heidelberg (09/2007)

Study type	Number of studies	Patients included	Patients randomised
Surgical RCTs	15	1277	1022
Pharmaceutical studies (Phase I – IV)	30	880	771
Observational studies	4	405	*
Total	49	2601	1793

* No randomisation is performed in observational studies

Table 3 gives an overview of key issues to be considered for organisation of CTCs and CSs based on the knowledge acquired by the SDGC and KSC during the last years. The different mentioned aspects should be addressed via specific Standardized Operating Procedures.

**Table 3:** Key issues to be considered for organisation of Clinical trial Centres (CTC) and Clinical Sites (CS).

	Clinical Trial Centre (CTC)	Clinical Site (CS)
Study selection	• Evaluation and decision making by transparent process according to FINER-Criteria [11] *F *Feasible *I *Interesting *N *Novel *E *Ethical *R *Relevant • Funding sources avaliable	• How many eligible patients can I get? (approx. 10 patients per year and trial are recommended) • Does the case money cover all costs? • Are the proposed interventions feasible at the institution? • Can patients be followed up according to the protocol?
Study conduction	• Adequate recruiting of CS (prior trial experience, sufficient patient populations) • Continuous supervision of Clinical Sites performance (e.g. monitoring, auditing) • Meetings prior to start of patient recruitment • Training of participating surgeons in methods of clinical Trials	• Standardized enrollment process (i.e. screening of all admitted patients, informed consent, randomisation) • Adherence to the study interventionsl and the national and international guidelines (e.g. GCP, Declaration of Helsinki) • Organization of follow up and documentation • Controlling of recruitment, treatment, and financial procedures
Networks and Partners	• Scientific societies • Governmental authorities (e.g. FDA) • National and international experts in trial methodology and the individual speciality • Funding organisations	• Ethics committee • Administration (contracts) • Departments collaborating in treatment of patients (e.g. anaesthesiology, radiology) • Institutions involved in required tests or delivery of study materials (e.g. laboratory, pharmacy) • IT Infrastructure
Required human resources	• Principal Investigator • Project manager • Biostatistician • Data manager • Monitor • Quality Assurance agent • IT Manager • Administrative agent	• Study physician • Study nurse

## Conclusion

The quantitiy and quality of sRCTs are substantially dependent on a functional trial infrastructure. The developments in Germany demonstrate that the concept of cooperating CTCs and CSs is a promising approach. Surgical departments are able to conduct sRCTs according to GCP guidelines within daily clinical routine, if they set up a CS staffed with at least one study nurse and one study surgeon. Furthermore, multi-centre sRCTs can be performed if collaboration of CTCs and CSs is institutionalised and the relevant scientific organisations are involved. Last but not least funding is an essential factor required for establishment of adequate infrastructure and the implementation of the projects. Consolidation and growth of the established institutions and network supported by sufficient funding will be essential for sustainable increase of sRCTs. In times of limited human recourses and financial constraints international collaborations will be a further step forward in order to enhance multi-centre sRCTs. The established trial infrastructure must not only ensure proper preparation and implementation of sRCTs, but also educate surgeons adequately about the principles of clinical trials.

## Competing interests

The author(s) declare that they have no competing interests.

## Authors' contributions

Conception and design of this article as well as analysis and interpretation of the data were contributions of NNR and CMS who also wrote the article. MKD, LF, MNW, PK, and MWB revised the manuscript.

All authors read and approved the manuscript.
